# A Self-Guiding Tool to Conduct Research With Embodiment Technologies Responsibly

**DOI:** 10.3389/frobt.2020.00022

**Published:** 2020-02-21

**Authors:** Laura Aymerich-Franch, Eduard Fosch-Villaronga

**Affiliations:** ^1^Communication Department, Pompeu Fabra University, Barcelona, Spain; ^2^eLaw - Center for Law and Digital Technologies, Leiden University, Leiden, Netherlands

**Keywords:** embodiment, responsible research & innovation (RRI), body ownership, ethics, virtual reality, social robots, embodiment technologies, avatars

## Abstract

The extension of the sense of self to the avatar during experiences of avatar embodiment requires thorough ethical and legal consideration, especially in light of potential scenarios involving physical or psychological harm caused to, or by, embodied avatars. We provide researchers and developers working in the field of virtual and robot embodiment technologies with a self-guidance tool based on the principles of Responsible Research and Innovation (RRI). This tool will help them engage in ethical and responsible research and innovation in the area of embodiment technologies in a way that guarantees all the rights of the embodied users and their interactors, including safety, privacy, autonomy, and dignity.

## Introduction

For some time now, there has been an increasing interest in the development of technologies that can couple the human body to a computer interface (Biocca, 1997). Over the years, technology has evolved to the point where it is possible to induce the illusion of embodiment (Madary and Metzinger, 2016) in a virtual (e.g., Slater et al., 2010) or a robotic avatar (e.g., Aymerich-Franch et al., 2017). Specifically, an extensive body of work in the area of virtual reality and social robots has repeatedly demonstrated that, when people embody a virtual or a robotic avatar, they experience body ownership over the body of that avatar (e.g., Slater et al., 2009; Aymerich-Franch et al., 2017) and self-location within its bodily boundaries (e.g., Lenggenhager et al., 2007; Slater et al., 2009). Crucially, during embodiment experiences, users experience the illusion that “what is apparently happening is really happening” (Slater, 2009). Hence, they respond to virtual agents, avatars (Garau et al., 2005), and threats (Slater et al., 2010) as if they were real.

Previous works have drawn attention to the importance of accounting for ethical issues in using immersive virtual reality (Southgate et al., 2017). However, the extension of the sense of self to the avatar during embodiment experiences (Aymerich-Franch, 2018) is a critical aspect that requires special ethical and legal consideration, principally, in light of potential scenarios involving physical or psychological harm caused to, or by, embodied avatars (Aymerich-Franch and Fosch-Villaronga, 2019; Aymerich-Franch et al., 2019).

The likely convergence of social networks and virtual reality represents one of the best examples of these likely scenarios. Potential threats to autonomy (i.e., the capacity to make uncoerced decisions) and privacy arising from this convergence have already been highlighted (O'Brolcháin et al., 2016). These threats, however, do not only apply to scenarios in which the technology reaches the final user, but also to research contexts. Avatar embodiment experiments are frequently used to recreate dangerous or stressful situations for the user to study human behavior and the fact that users may experience these situations as if they were real entails important ethical challenges (Pan and Hamilton, 2018).

Unfortunately, while the pace of technology development and their applied uses for research dramatically accelerate, the understanding of its implications does not follow in parallel. Thus, the literature falls short in reflecting on the legal and ethical implications of the development and use of embodiment technologies.

The lack of specific regulatory guidelines for embodiment technologies does not help either. Although a vast number of laws and norms might already apply to avatar embodiment, emerging technologies tend to fall into an “institutional void” (Hajer, 2003), challenging the understanding of which and how the regulations apply to a particular technology (Fosch-Villaronga, 2019). The lack of guidance in this respect challenges the legal certainty concerning what boundaries need to be respected, which rights users have, what obligations developers should abide by, and what consequences exist for non-compliance (Stilgoe et al., 2013; Fosch-Villaronga and Heldeweg, 2018; Fosch-Villaronga and Golia, 2019).

Our contribution attempts to dissipate the uncertainty that this scenario raises by creating a self-guiding tool based on the principles of Responsible Research and Innovation (RRI). This tool aims at helping researchers in the field of embodiment technologies engage in ethical and responsible research and innovation processes to develop, test, and implement these technologies guaranteeing the rights of the embodied users and their interactors, including the rights of safety, privacy, autonomy, and dignity.

RRI is an overarching concept that captures crucial aspects concerning what researchers can do to ensure that research and innovation have desirable outcomes (Stahl et al., 2014). It is often the case, however, that such good intentions struggle to translate into specific, practical, and widely adopted actions. The tool that we propose contributes to materialize the principles of RRI in the specific context of research and development of embodiment technologies to overcome this problem.

## Responsible Research And Innovation (RRI)

For the European Union, to help innovate responsibly and contribute to ensuring a desirable future for humanity translates into the Responsible Research and Innovation (RRI) framework (European Commission, 2012). The RRI approach provides a suitable framework to guide all the social actors involved in research and innovation (R&I) processes toward this aim. The European Commission (2019) defines RRI as “an approach that anticipates and assesses potential implications and societal expectations concerning research and innovation, intending to foster the design of inclusive and sustainable research and innovation.”

From the lens of RRI, the principles of anticipation, reflection, inclusion, responsiveness, and transparency typically guide R&I processes:

- Anticipation. Anticipation is about encouraging social actors involved in R&I processes to ask “what if” questions so that they envision contingency plans toward potential outcomes, build socially robust, risk-free research, and unveil hidden opportunities (Stilgoe et al., 2013).
- Reflection. Reflexivity encourages researchers to think mindfully about their work. Rethinking prevailing assumptions, values, and purposes in current R&I practices and activities may help raise awareness of the importance of framing issues, problems, and suggested solutions.
- Inclusion. The principle of inclusion is concerned with conducting research not only for society but *with* society and thus involving a wide range of stakeholders from the early stages of the R&I process “both for normative democratic reasons and to broaden and diversify the sources of expertise, disciplines, and perspectives” (Kupper et al., 2015).
- Responsiveness. RRI can reshape R&I processes in response to circumstances that no longer align with the continually evolving needs of society (Stilgoe et al., 2013). Responsiveness alludes to the flexibility and capacity to change R&I processes to ensure the research enforces public values.
- Transparency. Transparency encourages open-access dissemination of the results and conclusions, enabling this way public scrutiny and dialogue.

## Self-Guiding Tool to Conduct Research With Embodiment Technologies Responsibly

RRI promotes reflection upon the consequences of the outcomes of technology and fosters the incorporation of such reflections into the research and design processes. The five principles of inclusion, anticipation, reflection, responsiveness, and transparency that define RRI provide a suitable framework for conducting research and innovating responsibly in any area of R&I, including embodiment technologies. However, one of the most challenging aspects of being able to put into practice these principles is how to implement them in everyday R&I practices, accurately.

Following a basic coaching principle that *finding the right answers is about asking the right questions*, we provide a self-guiding tool with a series of critical questions inspired on Stilgoe et al. (2013), Kupper et al. (2015), and Stahl and Coeckelbergh (2016) concerning each of the five RRI principles (Table 1). Altogether, these questions work as a self-guidance tool that researchers and innovators can use to guide their R&I processes throughout all the stages, from the conception of the project to the final implementation or publication; and throughout all the dimensions, including the process itself, the product, the purposes, and the people involved (Stahl and Coeckelbergh, 2016).

**Table 1:** A self-guiding tool to conduct research with embodiment technologies responsibly.

**ANTICIPATION**
• What are the psychological, ethical, moral, and legal implications that the embodiment technology I am developing could bring? • Could someone use the embodiment technology that I am developing for unintended practices in my or other fields? Who and how? If so, what measures have I developed to mitigate this? • What are the risks and benefits of the embodiment technology that I am developing? How will they be distributed in society if the technology gets implemented? • If I am designing avatars that will interact with other avatars or real people, do I provide enough protection mechanisms to my users so that they can protect their avatars in case of assault? Which ones? • If I am working with physical avatars such as robots that could potentially harm someone in case of technical failure, have I considered emergency mechanisms so that the user can completely stop the action of the avatar or alternative control mechanisms to avoid causing harm to others or to the environment? Which ones? • Who needs to take responsibility if something goes wrong with the embodiment technology that I am developing? • Have I projected potential scenarios in which different aspects go wrong and have I determined who should take responsibility on each case? Which ones? • What aspects of the avatars that I am developing could make them not socially desirable? How can I change that?
**REFLECTION**
• Who could be negatively affected by my research with embodiment technologies? How can I change that? • How could the embodiment technology I am developing challenge the rights of future users? How can I avoid that? • Am I equipped with enough knowledge to identify and address the implications of embodiment technologies by myself? If not, what experts could help me and how? • Do I lead or participate in actions oriented at addressing concerns and fears regarding embodiment technologies for the general public? Which ones? Is there anything else I can do? • Do my results contribute to providing useful insights for developers regarding how to commercialize embodiment technologies safely for the society from an ethical point of view? How exactly? Is there anything else I can do to ensure that? • Do my results contribute to providing useful insights for regulators and legislators regarding how to regulate and legislate embodiment technologies? How exactly? Is there anything else I can do to ensure that? • Do I discuss the ethical implications of my results when I report them? • Have I conducted an actor analysis to understand which actors play an essential role for the embodiment technology that I am developing and identify people, industries, institutions, or organizations who can affect or are affected by the technology? • Have I sufficiently reflected on the benefits and risks of the embodiment technology before starting its development: does it honestly and positively contribute to society? How exactly? Is it aimed to resolve a societal challenge? Which one/s? • Have I organized discussion groups with the different stakeholders involved in the embodiment technology that I am working with to discuss potential ethical implications and create awareness of responsibility and accountability? • Am I open to receive criticism and consider skepticism about the embodiment technology or experiments using it and integrate it into my research/design process? What could I do differently to encourage more feedback from the relevant stakeholders? • Does my research with embodiment technologies have long-term consequences that could be potentially negative for society? How can I avoid them?
**INCLUSION**
• Am I familiar with the embodiment technologies that other researchers, companies, and start-ups are developing and the experimental work that other researchers are conducting with these technologies? What can I do to know them even better? • Who are the relevant stakeholders in the development of my research in embodiment technologies? Have I included them in the process? How exactly? • Could the virtual and robot avatars that I create cause gender, race, religious or age discrimination? How can I avoid this? • Do I respect the principle of diversity when I design avatars so that I have enough avatar choices that can represent well all potential participants or users and do not create conflict in terms of gender, race, ethnicity, religion, and other demographics? (e.g., I have a participant that wears a hijab in real life and does not feel comfortable embodying an avatar that does not, do I have the right type of avatar for her?) How could I increase the range of options to ensure that? • Have I talked directly to the stakeholders that my embodiment technologies target to enquire about how the technology could really improve their quality of life rather than making assumptions about it? (e.g., I am designing a robot avatar for persons with reduced mobility, but I actually never asked them directly if that is something that could be useful for them and how exactly would they want it) Who else should I talk to? • Do I engage the target of the embodiment technologies I design in my R&I processes throughout all the stages? How exactly? What else can I do to ensure that?
**RESPONSIVENESS**
• What do I need to do to ensure social desirability for the research I conduct with embodiment technologies? • What training am I receiving to conduct research with embodiment technologies responsibly? • If I encounter ethical conflicts, are there any barriers (e.g., economic interests) that prevent me from changing the course of the development of the embodiment technology? Which ones? What can I do to overcome them? • Is the technology of embodiment that I am developing prepared to evolve with the constantly evolving technological landscape? How exactly? What else can I do to prepare it better in this regard? • Is the technology of embodiment that I am developing responding clearly to current societal needs? To which ones specifically? • If I conduct experiments with embodiment technologies, are they meant to respond to societal needs and challenges? To which ones specifically?
**TRANSPARENCY**
• Are the motivations of the embodiment technology that I am developing transparent, honest, and geared toward the public interest? (e.g., I write a grant in which I argue that the embodiment technology will be useful for rescue operations just to get the grant, but I already know it will not be useful) • Am I sharing the results not only with the scientific community but also with the target of the embodiment technology? How am I doing so? • Am I openly sharing the uncertainties and limitations of the embodiment technology that I have developed with the full range of stakeholders as well as in my publications? All of them? (If this is something difficult for me, ask: What is the worst that could happen if I did so?) • Do I share the lessons learned of the research with embodiment technologies with my community (including the negative aspects)? How exactly do I do that?

By raising, reflecting, and answering these questions, researchers and developers will equip themselves with the necessary questions to further research with embodiment technologies responsibly. The tool will help researchers and developers integrate the reflections concerning the consequences of their work into their design processes and, hence, foster responsible technology, in line with societal needs and values.

While the tool specifically targets embodiment technologies, it can easily be adapted to other emerging technologies, hence, it is useful to a much wider community, including researchers from cyberpsychology, virtual reality beyond avatar embodiment, human-computer and human-robot interaction, affective computing, and other related fields.

## Discussion

Innovating is about creating and transforming the future. However, transforming the future does not necessarily mean for the better. Researchers might not always be able to foresee the potential negative impacts of their research on society. Users might also be more focused on the practical benefits they gain from employing the technology than on reflecting whether that is beneficial for them or not (Carr, 2011). As Parsons notes (Parsons, 2019), an unfortunate limitation of cyberpsychology literature is that “it rarely discusses the ways in which technologies are increasingly part of personhood or the ethical issues that result” (p. XVI-Preface). This work aims at mitigating this lack of reflection, at the same time, offering a tool that allows researchers to take action toward correcting potential bad practices.

The self-guiding tool to conduct research with embodiment technologies responsibly that we provide sets a series of questions taking into account the different principles of RRI to help researchers and developers steer the development of embodiment technologies into a desired and socially accepted direction.

The extension of the sense of self to the avatar implies that if the avatar is harmed, the embodied user experiences psychological harm as a result (Aymerich-Franch and Fosch-Villaronga, 2019). Also, it implies that, if the avatar causes harm due to technical failure, the users can falsely attribute responsibility to themselves, even if it is not their fault (Aymerich-Franch et al., 2019). A mindful reflection of the potential implications of embodiment technologies may prevent the occurrence of undesired outcomes such as the exacerbation of existing behaviors, sexual harassment of the avatar, or the wrong self-attribution of responsibility.

As McBride and Stahl (2014) highlight, RRI has developed at the governance level, but this does not ensure that practitioners will follow it, as governance procedures usually end up being considered as “hurdles to be jumped over and administrative rituals to be fulfilled.” In this respect, it is essential to promote, in parallel to actions at the governance level, institutional change to foster reflection on the consequences of the technology among researchers. To this end, the effective integration of all these reflections into the R&I process implies, as a first step, inviting researchers to ask themselves a series of questions focused on understanding whether the technological development that they carry out aligns with the RRI principles. The answer to these questions may trigger the researcher to take action (e.g., by establishing dialogues with different stakeholders or by seeking further guidance and additional training to equip themselves with the adequate tools to better understand how to frame possible concerns and how to mitigate them).

In this paper, we have taken one step forward toward bridging the gap between conceptual and applied RRI by translating the general principles of the RRI framework into a practical tool for conducting research with embodiment technologies. This self-guiding tool is intended to help researchers give careful thought to the consequences of the technology they develop in an anticipatory, inclusive, reflective, responsive, and transparent manner. That said, the tool we present is by no means to replace regulatory and ethical compliance processes, but an invitation to reflect deeply and consciously about the societal implications of work with embodiment technologies. It aims to the realization of the RRI goals by creating a practical, integrative, reflective mechanism geared toward addressing embodiment technologies' societal implications.

To conclude, innovating in a responsible manner contributes to ensuring a desirable future for humanity. By carrying out the self-assessment, we hope researchers in this field will become more sensitive to how essential it is to steer their research in a responsible direction. On most of the occasions, this process will push the boundaries toward a more interdisciplinary, integrative, and thoughtful model of conducting research that may also be more beneficial for the society in the long run.

## Author Contributions

All authors listed have made a substantial, direct and intellectual contribution to the work, and approved it for publication.

### Conflict of Interest

The authors declare that the research was conducted in the absence of any commercial or financial relationships that could be construed as a potential conflict of interest.
